# Determinants of infant nutritional status in Dabat district, North Gondar, Ethiopia: A case control study

**DOI:** 10.1371/journal.pone.0174624

**Published:** 2017-03-27

**Authors:** Amarech Asratie Wubante

**Affiliations:** West Gojjam Zonal Health Department, Finoteselam, Ethiopia; Centre Hospitalier Universitaire Vaudois, FRANCE

## Abstract

**Background:**

Malnutrition is the top cause of global burden of disease, disability and mortality among infants. Over two-thirds of deaths of children globally occur during the first year of life (infancy). Malnutrition among infants is substantially high in Ethiopia. Therefore, this study is aimed to assess determinants of infant nutritional status.

**Methods:**

A community based nested case-control study was conducted from February to June 2013 in Dabat district. A total of 80 cases and 320 controls (1:4 ratios) were studied. Relevant data was extracted from the community based survey data set. Anthroplus software was used to identify cases and controls. Determinants of infant nutritional status were identified using multivariate analysis.

**Results:**

Among the total of 80 cases and 320 controls, more than half (52.5%) of the cases and the controls (53.8%) were males and females, respectively. Breast Feeding (BF) was started immediately after birth in only 43.8% of the cases. Nearly 94% of the mothers of the cases had no breast feeding information as part of Ante Natal Care (ANC) follow up. Maternal age (AOR: 0.29; 95% CI: 0.11–0.76), having radio (AOR: 0.43; 95% CI: 0.22–0.82), lack of toilet facility (AOR: 2.24; 95% CI: 1.16–4.33), deprivation of colostrum (AOR: 1.76; 95% CI: 1.01–1.06) and method of complementary feeding (AOR: 2.82; 95% CI: 1.33–5.99) were associated with wasting.

**Conclusions:**

This study has found that inappropriate infant feeding; nutritional information gap and lack of toilet facility as significant predictors of malnutrition. Hence, joint interventions, including counseling of mothers about benefits of colostrum feeding and use of appropriate feeding method, toilet utilization and mass media such as radio possession, are needed to address the problem in Dabat district.

## Background

Malnutrition is the top cause of global burden of disease, disability and mortality among infants and children. One hundred sixty one million children under age 5 are too short for their age (stunted), 51 million don’t weigh enough for their height (wasted), and 42 million are overweight; none of these children are growing healthily [1]. Malnutrition is a factor for an estimated 53% of all childhood deaths globally [2]. Over two-thirds of these deaths, which are often associated with inappropriate feeding practices, occur during the first year of life (infancy) [3].

Ethiopia is one of the developing countries where malnutrition remains a major public health problem. According to the 2011EDHS data, wasting among 9–11 months aged infants was 19%, and 10% of babies below six months are underweight [4]. Furthermore, studies in Ethiopia showed poor infant feeding practice, poor socio-economic background and nutritionally inadequate diet that contributed more for severe acute malnutrition among infants [5, 6].

Infants are particularly vulnerable for malnutrition during the transition period due to a number of factors like suboptimal weaning and inappropriate complementary feeding [7], social factors such as deprivation of maternal education [8], and health factors related to infectious diseases like diarrhea [9]. Nutritional deficits acquired in the early stages of life (infancy) are difficult to reverse later [3]. Hence, interventions before birth (targeting pregnant women) and appropriate infant and young child nutrition can make the largest difference.

The Ethiopian Health Sector Development Program (HSDP) IV has been launched to improve the nutritional status of mothers and infants through enhanced outreach strategy with targeted supplementary food, health facility nutrition services, community-based nutrition, micronutrient interventions and essential nutrition actions/integrated infant and young feeding counseling services [10]. However, still malnutrition among infants is substantially high [4]. Therefore, the exact determinant factors needs be well studied to prevent infant malnutrition.

## Methods

### Study design and area

A community based nested case control study design was used to assess determinants of infants’ nutritional status. The study was conducted between February and June 2013 at Dabat Research Center, Northwest Ethiopia. Dabat is bordered by Wegera in the South, Tach Armachiho in the West, Tegeda in the Northwest, and Debarq in the Northeast. Based on the 2007 national census conducted by the Central Statistical Agency (CSA), the district has a total population of 145,509 [11]. It covers an area of 1,187.93 square kilo meters, and has a population density of 122.49 persons per square kilometer.

### Study subjects, sample size and sampling procedure

All infants that were enrolled in the infant mortality prospective follow-up study at the Dabat research center were our study population. Infants, who had relevant and complete data, were nested as study subjects. A total of 400 infants (80 cases and 320 controls; 1:4) were studied. The sample size was determined by considering family size was significant predictors of infant nutritional status and proportion of infants of large family size among the controls 25.5% [12], the level of significance (α) = 0.05, the power of the test (1-β) = 80%, and the case to control ratio(r) = 1:4, OR = 1.96. It was calculated using Open Epi version 2 software. All infants having measurement (weight and height) at six months and 12 months were considered and selected consecutively from the dataset.

### Data collection procedures and quality assurance

Data extraction tool designed to collect necessary information from the database developed by Dabat Research Center was used (S1 File). The data was extracted from the database previously collected for child survival cohort study. Anthropometric measurements of infant weight, height and mid upper circumference using standardized methods of the World Health Organization (WHO) were collected from the database. Weight of the infant was previously measured in kilo gram and recumbent length in centimeters on a length board. For this study, a new questionnaire was again developed based on variables of interest to extract relevant data from the given database found in the child survival cohort. Required data was then extracted and new data set was created.

After editing and cleaning, anthropometric data were imported into WHO anthroplus software to calculate Weight for Age (WAZ), Weight for Height (WHZ) and Height for Age (HAZ). Infants that had WAZ, HAZ and WHZ values below -2 Standard Deviation (SD) from the median of the reference population were considered as malnourished for underweight, stunting and wasting, respectively [13]. Infants that were underweight or stunted or wasted were considered as cases. Only complete and relevant data were used to ensure data quality.

### Data management and analysis

Data were imported into SPSS version 16 for further analysis. Frequencies and cross tabulations were calculated to describe cases and controls. Variables having p-value < 0.2 in the Bi-variate analysis were entered into multiple logistic regressions to control the effect of confounders.

Significant association between study variables and interpretation of data was done using odds ratio (OR) and 95% confidence interval. Those variables having significant association at 95% CI were considered as determinant factors for infant nutritional status.

### Ethical consideration

This study was reviewed and approved by the ethical review board of the institute of Public Health, University of Gondar. Acceptance letter was obtained from the Dabat Research Center to collect the data from the database. Informed consent of the infant’s parents or guardian was not found since we used the database from Dabat Research Center. Only the author had the right to access data in order to keep confidentiality.

## Results

### Socio-demographic characteristics of the participants

Among the total of 80 cases and 320 controls, more than half (52.5%) of the cases and the controls (53.8%) were males and females, respectively. About 70% of the cases and 68% of the controls were from mothers aged 20–34 years. Nearly 94% of the mothers of the cases were house wives. Thirty percent of the cases had the lowest wealth indexes (Table 1).

**Table 1:** Socio-demographic characteristics of infants in Dabat District, 2013, (n = 400).

Characteristics	Category	Cases Number (%)	Controls Number (%)
Sex of the child	Male	42 (52.5)	148(46.2)
	Female	38 (47.5)	172(53.8)
Maternal age	19 and less years	13 (16.2)	30 (9.4)
	20–34 years	56 (70.0)	219 (68.4)
	Above 34 years	11 (13.8)	71 (22.2)
Maternal literacy	No	56 (70.0)	218 (68.1)
	Yes	24 (30)	102 (31.9)
Mother’s main occupation	House wife	75 (93.8)	289 (90.3)
	Employed	2 (2.5)	6 (1.9)
	Jobless	3 (3.8)	9 (2.8)
	Others	0 (0)	16 (5.0)
Religion	Orthodox	79 (98.8)	314 (98.1)
	Muslim	1 (1.2)	6 (1.9)
Paternal literacy	No education	42 (52.5)	184 (57.5)
	Read and write	38 (48.5)	136 (42.5)
Wealth index	Lowest	23 (28.8)	104 (32.5)
	Medium	24 (30.0)	103 (32.2)
	Highest	33 (41.2)	113 (35.3)
Residential area	Urban	24 (30.0)	74 (23.1)
	Rural	56 (70.0)	246 (76.9)
Own radio	No	20 (25.0)	59 (18.4)
	Yes	60 (75.0)	261 (81.6)
Number of under five children	<3	78 (97.5)	308 (96.2)
	≥3	2 (2.5)	12 (3.8%)

### Clinical profiles and environmental sanitation practices

Birth interval of above 24 months in the cases were 52 (65%), and 241 (75.3%) in the controls. Majority of the cases (98.8%) and the controls (98.8%) had no fever in the last two weeks. Toilet facility was lacked by 62 (77.5%) of the cases and 213 (66%) of the controls. More than 57% of the family of the cases had unprotected water supply (Table 2).

**Table 2:** Clinical and environmental profiles of infants at Dabat district, 2013, (n = 400).

Variables	Category	Cases Number (%)	Controls Number (%)
Birth interval	0–24 month(s)	28 (35.0)	79 (24.7)
	Above 24 months	52 (65.0)	241 (75.3)
Birth order	First	17 (21.2)	44 (13.8)
	2–4	37 (46.2)	136 (42.5)
	Five and above	26 (32.5)	140 (43.8)
Main water source	Protected	34 (42.5)	126 (39.4)
	Unprotected	46 (57.5)	194 (60.6)
Use of toilet facility	Yes	18 (22.5)	107 (33.4)
	No	62 (77.5)	213 (66.6)
Solid waste disposal system	Proper	2 (2.5)	10 (3.1)
	Improper	78 (97.5)	310 (96.9)
Fever in the last two weeks	No	79 (98.8)	316 (98.8)
	Yes	1 (1.2)	4 (1.2)
Diarrhea in the last 2 weeks	No	74 (92.5)	289 (90.3)
	Yes	6 (7.5)	31 (9.7)

### Infant feeding practices

Breast Feeding (BF) was started immediately after birth in 43.8% of the cases. Ninety percent of the cases had weaning time at six months. More than half of the mothers preferred porridge as weaning food both in the cases and controls group. Exclusive breast feeding was used by 90% of the cases and 84.4% of controls (Table 3).

**Table 3:** Practices of infant feeding in Dabat District, 2013, (n = 400).

**Variables**	**Category**	**Cases Number (%)**	**Controls Number (%)**
Initiation time of breast feeding	Immediately	35 (43.8)	127 (39.7)
	1–24 hour(s)	28 (35.0)	122 (38.1)
	After 24 hours	17 (21.2)	71 (22.2)
Colostrum feeding	Given to the baby	57 (71.2)	186 (58.1)
	Removed	23 (28.8)	134 (41.9)
Pre-lacteal feeding	No	54 (67.5)	200 (62.5)
	Yes	26 (32.5)	120 (37.5)
Exclusive breast feeding	<6 months	8 (10.0)	50 (15.6)
	≥6 months	72 (90.0)	270 (84.4)
Time of imitation of additional food	Below 6 months	5 (6.2)	14 (4.4)
	At 6 months	72 (90.0)	273 (85.3)
	Above 6 months	3 (3.8)	33 (10.3)
Method of feeding	Spoon	13 (16.2)	100 (31.2)
	Cup feeding	30 (37.5)	85 (26.6)
	Hand feeding	34 (42.5)	124 (38.8)
	Bottle feeding	3 (3.8)	11 (3.4)

### Health care utilization practice

Only 5% of the cases and 4.7% of the controls were taken to the health institution during illness. More than 81% of the mothers both in the cases and controls had no iron supplementation. About 98% of the mothers of cases and controls did not take de-worming. Nearly 94% of the mothers of the cases had no breast feeding information as part of ANC follow up (Table 4).

**Table 4:** Health care utilization practice of infants and mothers in Dabat District, 2013 (N = 400).

Variables	Category	Cases Number (%)	Controls Number (%)
Infant took to health facility during illness	No	76 (95.0)	305 (95.3)
	Yes	4 (5.0)	15 (4.7)
ANC follow up	No	57 (71.2)	215 (67.2)
	Yes	23 (28.8)	105 (32.8)
Maternal nutrition information	No	70 (87.5)	254 (79.4)
	Yes	10 (12.5)	66 (20.6)
Taking de-worming drugs	No	78 (97.5)	314 (98.1)
	Yes	2(2.5)	6(1.9)
Breast feeding information	No	75 (93.8)	276 (86.2)
	Yes	5 (6.2)	44 (13.8)
Take iron during last pregnancy	No	65 (81.2)	263 (82.2)
	Yes	15 (18.8)	57 (17.8)
Place of delivery	Home	53 (66.2)	232 (72.5)
	Health facility	27 (33.8)	88 (27.5)

ANC: Ante Natal Care

### Determinants of nutritional status of infants

In the multiple logistic regression analysis, deprivation of colostrum, having radio, use of toilet facility, method of complementary feeding and maternal age were significantly associated with wasting. In brief, those who had deprivation of colostrum were about two times (AOR: 1.76; 95%CI: 1.01–3.06) more likely to be wasted. Infants from the families who had a radio were 0.43 times (AOR: 0.43; 95%CI: 0.22–0.82) more likely to have better infant nutrition as compared to those who did not have. Those infants who had family with no toilet facility were 2.42 times (AOR: 2.24; 95%CI: 1.16–4.33) more malnourished. Cup feeding was almost three times (AOR: 2.82; 95%CI: 1.33–5.99) more to cause wasting compared to spoon feeding. Those infants who had mothers aged >34 years were 0.29 times (AOR: 0.29; 95%CI: 0.11–0.76) less likely to be malnourished as compared to those that had mothers aged 19 years or less (Table 5).

**Table 5:** Determinants of infant nutritional status in Dabat district, 2013 (n = 400).

Variables	Categories	Nutritional status		Crude OR (95% CI)	Adjusted OR (95% CI)
		Cases	Controls		
Colostrum feeding	Removed	57	186	1.79 (1.05–3.04)	1.76 (1.01–3.06)
	Given	23	134	1	1
Own radio	No	20	59	1	1
	Yes	60	261	0.68 (0.38–1.21)	0.43 (0.22–0.82)
Have toilet facility	Yes	18	107	1	1
	No	62	213	1.73 (0.98–3.07)	2.24 (1.16–4.33)
Nutrition information	No	70	254	1	-
	Yes	10	66	0.55 (0.27–1.13)	-
Breast feeding information	No	75	276	1	1
	Yes	5	44	0.42 (0.16–1.09)	0.40 (0.14–1.14)
Time of additional food initiation	<6 months	5	14	1	
	At 6 months	72	273	0.74 (0.26–2.12)	-
	> 6months	3	33	0.26 (0.05–1.21)	-
Feeding methods	Spoon	13	100	1	1
	Cup	30	85	2.72 (1.33–5.53)	2.82 (1.33–5.99)
	Hand	34	124	2.11 (1.06–4.21)	1.50 (0.70–3.20)
	Bottle	3	11	2.10 (0.52–8.52)	1.428 (0.33–6.28)
Birth interval	0–24 month(s)	28	79	1	-
	> 24 months	52	241	0.61(0.36–1.03)	-
Birth order	First	17	44	1	
	2–4	37	136	0.70 (.36–1.37)	-
	≥5	26	140	0.48 (0.24–0.97)	-
Maternal age	≤19 years	13	30	1	1
	20–34 years	56	219	0.59 (0.29–1.21)	0.59 (0.28–1.24)
	>34 years	11	71	0.36 (0.14–0.89)	0.29 (0.11–0.76)

**Note:** Hosmer and Lemeshow Test; X^2^ = 5.055; P. value = 0.752

**Abbreviations:** COR: Crude Odds Ratio; AOR: Adjusted Odds Ratio; CI: Confidence Interval

## Discussion

According to the 2011EDHS report, infant malnutrition is a public health concern in Ethiopia [4]. Some of the short and long-term consequences of infant malnutrition include an increased susceptibility to diarrhea and respiratory infection, faltered growth, impaired mental development, death and attention deficit at middle adult hood [4, 14]. Therefore, strategies involving only the screening and treatment of the severely malnourished will do little to address this impact.

In this study, infants that fed colostrum had better nutritional status than those who didn’t. Similarly, association between malnutrition and was feeding the colostrum reported from studies conducted in west Gojjam [15] and in India [7]. This may be because of the colostrum is very rich in proteins, carbohydrates, vitamin A, and sodium chloride, and contains various immune components to fight against viral and bacterial infections [16–18].

This study found that infants who had mothers aged >34 years were less likely to be malnourished. Better baby caring experience developed by older mothers may attribute this result. In line with this study, age of mothers >32 years was stated as facilitating factor of better nutritional status in Avon [19].

In this study, infants from the families who had a radio were more likely to have better infant nutrition as compared to those who did not have. A recent study also showed that exposure to mass media promotes health-related behaviors, including nutrition [20]. Radio stations are regarded as reliable sources of advice on general child care matters, broadcasted general health and nutrition that facilitated the individual counseling, and optimal complementary feeding practices [10, 21].

In the present study, lack of toilet facility significantly increased the risk of malnutrition about two times. This finding is in line with studies done in Butajira [5]. It is possibly explained by inadequate hygiene and sanitation that could lead to dietary contamination and frequent infections, which further impairs infant’s nutritional status [9, 22, 23].

Method of feeding was significantly associated with infants’ nutritional status. Infants that had cup feeding practices were almost three times more likely to have malnutrition compared to those who had spoon feeding. This finding is similarly reported in west Gojjam [15] although a contradicting report documented that cup feeding infants were at risk of aspiration pneumonia, physiological instability and poor weight gain when used improper techniques [5].

As I used secondary data source, limitation, data of some variables that may have important input in determinants of infant nutritional status were not found.

## Conclusions

This study has found that maternal age above 34 years, having radio, colostrum feeding, method of feeding and having toilet facility were found to have significant association with wasting. Hence, joint interventions are needed to address the problem of the communities which may include improving the housing conditions in the village by constructing proper toilets and latrines, provision of pure water supply, and mass media such as radio possession. Health education is crucial to emphasize personal hygiene and hand washing. Counseling of mothers about benefits of colostrum feeding and use of appropriate feeding method to their new born should be practiced well in Dabat district.

## Supporting information

S1 File Data extraction tool. (DOCX)

## Abbreviations

ANC: Ante Natal Care
BF: Breast feeding
EDHS: Ethiopian Demographic and Health Survey
HAZ: Height for Age
HSDP: Health Sector Development Program
SD: Standard Deviation
WAZ: Weight for Age
WHO: World Health Organization
WHZ: Weight for Height
