# Supplementary material for: Determinants of infant nutritional status in Dabat district, North Gondar, Ethiopia: A case control study
Source: PLoS One. 2017 Mar 27;12(3):e0174624. doi: 10.1371/journal.pone.0174624 (PMC5367808; doi:10.1371/journal.pone.0174624)
Supplement: S1 File — (DOCX) [file pone.0174624.s001.docx]

## Data extraction tool

General instruction: this extraction tool is used to identify determinants of infant nutritional status data from the database. For each question, there are response column and is code accordingly in front of it.

Infant Code number:________________________

Part I. Socio- demographic characteristics of mother (guardian) and father

| No. | Questions | Response options | Skip |
| --- | --- | --- | --- |
| 1 | Sex of infant | 1. Male 2. Female |  |
| 2 | Age of the mother at birth of this baby | ____ years |  |
| 3 | Educational status of the mother | 1.unable to read and write  2.informal education 3.Primary  3.Secondary  4.Higher  5.Don’t know |  |
| 4 | What is the mother’s religion? | 1. Orthodox 2. protestant 3. catholic 4. Moslem 5. Others |  |
| 5 | What is your current place of residence? | 1. Rural 2. urban |  |
| 6 | Mother’s occupation? | 1.Student  2.farmer  3.private  4. regular employ  5. contract employ  6.merchant  7. House servant.  8.Jobless  9.pension  10. hand cape  11.others |  |
| 7 | What was your husband or the father of the baby educational status | 1.unable to read and write  2.informal education  3.primar  4. Secondary  5.Higher 6.Don’t know |  |
| 8 | What was your husband/ baby’s father occupation? | 1.student  2.farmer  3.private  4. regular employ  5. contract employ  6.merchant  7.house servant  8.Jobless  9.Pension  10. hand cape  11.others |  |
| 9 | How many under five children do you have? | ______ |  |
| 10 | HH Wealth index | **1.** Lowest  2. Middle  3. Highest |  |

**Part II**. **Infant character**

| 11 | Birth day | _____ |  |
| --- | --- | --- | --- |
| 12 | The birth order of this baby | ____ |  |
| 13 | The birth interval between this baby and older sibling | ____month |  |
| 14 | Did the baby have diarrhea within the last 2 weeks (watery/bloody loosen stool more than 3 time a day) | 1.Yes  2.No  3.I do not know |  |
| 15 | Did the baby have febrile illness within the last 2 weeks (watery/bloody loosen stool more than 3 time a day) | 1. Ye 2. No 3. I do not know |  |

**Part III:** **Anthropometric measurement of the infant**

| 16 | Infant’s weight | ______ Kilo grams. |
| --- | --- | --- |
| 17 | Infant’s recumbent length | ______ cent meter |
| 18 | Infant’s upper arm circumference | ______ cent meter |

**Part IV. Infant care and feeding practice**

| 19 | Has the infant ever breast fed? | 1. yes  2.no  3.unknown |  |
| --- | --- | --- | --- |
| 20 | If not for the above question, what was the reason? | 1. Mother was sick/weak  2. baby was sick/weak  3. breast problem 4. breast have no enough milk  5. Mother is so busy 6. Baby wasn’t interested  7. Mother is died 8. Other |  |
| 21 | After you gave birth when did you initiate breast feeding? | 1.immediately 2.1-24 hours  3. After 24 hours 4. I don’t remember |  |
| 22 | What did you do on the colostrums? | 1. Given to the baby 2. Discarded |  |
| 23 | Did you feed the baby other liquid food within three days of birth? | 1. yes  2. no  3. I don’t know |  |
| 24 | How long did you breast feed your baby exclusively? | 1.__hours  2. ___days  3. __months  4. I don’t know |  |
| 25 | Is the baby on breast feeding now? | 1. Yes 2. No |  |
| 26 | If you stop breast feeding, how long did you lactate? | 1._day  2.__ month  3.I don’t know |  |
| 27 | Had the baby bottle feeding yesterday? | 1. Yes 2. No |  |

**Part V. Health care service utilization**

| 28 | How long does it take from your home to the nearby health facility on foot? | 1.___minuet/hour 2. I don’t know |  |
| --- | --- | --- | --- |
| 29 | Where did you have ANC during your last pregnancy? | 1.I hadn’t ANC 2. public hospital  3. Private hospital 4. private health center  5. Public clinic 6. private clinic  7. health post 8.NGos health center |  |
| 30 | Did you take anemia preventive drug (iron foliate tablet) during you last pregnancy? | 1. yes  2. no |  |
| 31 | Did you take de worming drugs during your last pregnancy? | 1. yes  2. no |  |
| 32 | Where did you delivered your last child? (if in health facility mention its name) | 1. My home 2. My mother home 3. Public hospital 4. public clinic/health center  5. Private hospital 6. private clinic |  |
| 33 | Did you have visual problem at night during your last pregnancy? | 1. yes  2. no  3.not known |  |
| 34 | Did you take the baby to health facility when he had diarrhea? | 1. yes  2. no |  |
| 35 | The amount of food the baby was taking when he had diarrhea None | 1.Less that  2.the same  3.More than before 4.very small  5.not known |  |
| 36 | Did you take the baby to health facility when he had febrile illness? | 1. yes  2. no  3. I don’t know |  |
| 37 | Did the baby take treatment from health facilities to malaria or other febrile illness | 1.yes  2.no  3.I don’t know |  |
| 38 | Did the baby take treatment outside of health facilities to malaria or other febrile illness? | 1.yes  2.no  3. I don’t know |  |
| 39 | Is the baby vaccinated? | 1. yes  2. no |  |
| 40 | Is the baby vaccinated on its fore leg? | 1. yes  2. no |  |
| 41 | If yes, how many times? | ____ |  |
| 42 | Did the baby take vaccine orally? | 1. yes  2. no |  |
| 43 | If yes, how many times? | _____ |  |
| 44 | How many times did the baby take vaccine orally | _____ |  |
